# Thoracic epidural analgesia prolongs postoperative QT interval on electrocardiogram in major non-cardiac surgery: a randomized comparison and a prospective cohort analysis

**DOI:** 10.3389/fphar.2023.936242

**Published:** 2023-05-19

**Authors:** Kotaro Hori, Shogo Tsujikawa, Mika Egami, Sayaka Waki, Ryota Watanabe, Hideki Hino, Tadashi Matsuura, Takashi Mori

**Affiliations:** ^1^ Department of Anesthesiology, Osaka Metropolitan University Graduate School of Medicine, Osaka, Japan; ^2^ Central Laboratory, Osaka Metropolitan University Graduate School of Medicine, Osaka, Japan; ^3^ Department of Anesthesiology, Osaka Rosai Hospital, Osaka, Japan

**Keywords:** anesthesia, epidural analgesia, levobupivacaine, electrocardiogram (ECG), QT interval

## Abstract

**Introduction:** Prolongation of QT interval on electrocardiogram can be associated with perioperative lethal arrhythmia. Epidural analgesia is a commonly used modality to relieve surgical pain by blocking sensory nerves, which also blocks the autonomic nervous system and can affect QT interval. Since patient monitoring becomes much less frequent after surgery than intraoperative period, we investigated the effects of epidural analgesia on postoperative QT interval with a randomized clinical trial and a prospective cohort study.

**Methods:** In a randomized study, we assigned 60 patients undergoing thoracic epidural analgesia to an epidural analgesia or no-epidural analgesia group, in which 3 ml/h of 0.25% epidural levobupivacaine (7.5 mg/h) was administered only in the epidural analgesia group during surgery. The primary outcome was the postoperative heart rate-corrected QT interval. In a prospective cohort study, patients were assigned to receive 5 ml/h epidural levobupivacaine (12.5 mg/h). The plasma concentration of levobupivacaine was measured using liquid chromatography-mass spectrometry.

**Results:** The median postoperative corrected QT interval interval with 3 ml/h epidural levobupivacaine was significantly longer than that without epidural analgesia. Using multiple regression analysis for the factors known to affect postoperative corrected QT interval interval, epidural analgesia was found to be an independent variable for prolongation, and the mean difference of the corrected QT interval interval with or without epidural analgesia was 23 ms after adjustment. The median plasma concentration of levobupivacaine at the end of surgery was 164 ng/ml with 3 ml/h epidural levobupivacaine, and the correlation coefficient to the postoperative corrected QT interval interval was 0.14, showing a not significant correlation. A prospective cohort study showed that 5 ml/h epidural levobupivacaine significantly prolonged postoperative corrected QT interval interval compared to preoperative baseline. The median plasma concentration of levobupivacaine was 166 ng/ml with 5 ml/h, the correlation coefficient of which showed no significant correlation.

**Conclusion:** Thoracic epidural analgesia could enhance postoperative corrected QT interval prolongation after general anesthesia. The mechanism is possibly caused by blocking neighboring or part of the cardiac sympathetic nerves, rather than by systemic effects of epidurally administered levobupivacaine.

**Clinical trial number:** UMIN000013347 for the randomized study and UMIN000041518 for the prospective cohort study, which were registered at University hospital Medical Information Network Center.

## Introduction

Torsade de pointes (TdP) is a polymorphic ventricular tachycardia, which is a rare but potentially lethal arrhythmia (Drew et al., 2010; Roden, 2016; Locati et al., 2017). Although the incidence of perioperative TdP is not well established due to lack of continuous ECG monitoring or possible less publications of the undesired complication, more than 40 cases of perioperative TdP have been reported over the past 40 years (Johnston et al., 2013). The incidence might be low, but it could result in mortality and thus should be considered with caution. A well-known cause of this arrhythmia is prolongation of cardiac repolarization, which can be identified by QT interval on electrocardiogram (ECG). Surgical patients are exposed to several conditions conducive to abnormal repolarization, such as systemic stress response, hypothermia, electrolyte disturbance, and several perioperative drugs known to affect the QT interval (Owczuk et al., 2012; Staikou et al., 2014; O'Hare et al., 2018). Previous studies showed that postoperative prolongation of the heart rate-corrected QT interval (QTc) is common after general anesthesia in major non-cardiac surgery (Nagele et al., 2012; Duma et al., 2016).

Neuraxial block, by administration of local anesthetics into the epidural or intrathecal space, blocks the autonomic nervous system in addition to sensory nerves and can affect cardiac repolarization (Taniguchi et al., 1997). Cardiac sympathetic nerves, which extend from thoracic vertebrae 1–4 (Th 1–4), are known to play important roles in controlling electrophysiological properties of the heart. It has been reported that a high level of thoracic epidural block, which can completely block cardiac sympathetic nerves with the catheter inserted at Th 1–2 or 2–3, or cardiac sympathetic denervation by surgery successfully reduced arrhythmic events with QTc shortening in patients with long QT syndrome (Schwartz et al., 2004; Bourke et al., 2010). QTc shortening by epidural block to the level of Th 1 was also observed in surgical patients without severe systemic diseases (Owczuk et al., 2009). However, in sharp contrast to this, a lower neuraxial block to the level of Th 10 or in the median maximum level of Th 4 with spinal anesthesia was shown to prolong the QTc interval rather than shorten it (Owczuk et al., 2005; Dogan et al., 2014). In these studies, reflective stimulation in unblocked cardiac sympathetic nerves is considered to be a potential cause for QTc prolongation.

Epidural catheters in standard perioperative settings are inserted at the middle or low thoracic level, not at the high level, and are often used as modality of perioperative analgesia combined with general anesthesia. Therefore, we hypothesized that epidural analgesia in standard clinical settings may enhance postoperative QTc prolongation after general anesthesia. Since patient monitoring becomes much less frequent after surgery than intraoperative period, the postoperative QTc interval is one of the most important outcomes to evaluate perioperative cardiac repolarization with epidural analgesia, but has not been examined in detail (Owczuk et al., 2012; Staikou et al., 2014). To test the hypothesis, we designed a randomized study in patients scheduled for thoracic epidural analgesia combined with general anesthesia. The postoperative QTc interval with 3 ml/h epidural levobupivacaine was compared to that without epidural drug administration. To further evaluate the dose-dependent effect of levobupivacaine, a prospective cohort study was performed with 5 ml/h epidural levobupivacaine during surgery.

## Methods

### Design, patients, and procedures (randomized comparative study)

This was a single-center, double (patient and assessor)-blinded, randomized comparative study, which was approved by the Osaka City University Graduate School of Medicine Institutional Review Board (Osaka, Japan), and was registered prior to patient enrollment at University hospital Medical Information Network Center (UMIN000013347, Principal investigator: Tadashi Matsuura, Date of registration: 5 March 2014). All patients provided written informed consent after being provided a complete description of the study protocol.

We enrolled 60 patients who were scheduled for general anesthesia with thoracic epidural analgesia during major non-cardiac surgery. Patients with atrial fibrillation or those classified as ASA (American Society of Anesthesiologists) physical status greater than II were excluded. Patients were randomly allocated to two groups in a 1:1 ratio: an “Epidural Analgesia” group and a “No Epidural Analgesia” group, by using computer-generated random numbers. In both groups, an epidural catheter (Arrow^®^ FlexTip Plus^®^ Epidural Catheter; Teleflex, Morrisville, NC, United States) was inserted before induction of general anesthesia, but the epidural drug was administered only in the epidural analgesia group during surgery. A test dose of 1% lidocaine (1.5 mL) with epinephrine (1:100,000) was used to confirm epidural catheter insertion. The baseline QTc interval was recorded before the epidural tap, and postoperative QTc was recorded at the end of surgery following tracheal extubation. The level of the thoracic vertebra of the epidural tap was selected at the discretion of the attending anesthesiologist, and this level was confirmed by chest radiography after surgery.

### Study outcomes

The postoperative QTc interval was used as the primary outcome to evaluate the potential risk of perioperative arrhythmogenicity. The data were first analyzed using an unadjusted comparison of the postoperative QTc interval between the two groups. To evaluate the influence of other perioperative factors that have previously been shown to affect the postoperative QTc interval (Nagele et al., 2012), multiple linear regression models were applied with the following variables: age, sex, dose of sevoflurane, and dose of ephedrine. The incidence of perioperative fatal arrhythmias was determined by medical record review until the end of epidural catheters use.

### Electrocardiogram recordings

In the operating room, patients were fitted with a standard real-time three-lead electrocardiogram (M1678A; Royal Philips, Amsterdam, Netherlands), and the II lead was recorded throughout the operation in a server storage system. In the epidural analgesia group, 4 ml of 0.25% (10 mg) levobupivacaine was administered through the epidural catheter after induction of general anesthesia, followed by continuous administration at 3 ml/h of 0.25% levobupivacaine (7.5 mg/h). No other epidural drugs were administered during surgery. In the no epidural analgesia group, epidural catheter was not used throughout the operation with the exception of the epidural test dose. Epidural analgesia in this group was started after recording the postoperative ECG to measure the QTc interval.

In all patients, no anesthetic premedication was administered. Anesthesia was induced with propofol, and maintained with sevoflurane. Fentanyl and remifentanil were used as opioids, and rocuronium was used as a neuromuscular blockade, which was reversed by sugammadex prior to extubation. Perioperative drugs other than epidural levobupivacaine were used at the discretion of the attending anesthesiologist. The plasma concentration of electrolytes was measured at the end of the surgery using blood gas analyzer (ABL 825; Radiometer, Copenhagen, Denmark). Body temperature was monitored at the urinary bladder (BARD Lubri-Sil Foley catheter; BD, Franklin Lakes, NJ, United States).

### Plasma concentration of levobupivacaine

To assess the systemic effects of epidural levobupivacaine on patients’ QTc interval, we measured the plasma concentration of levobupivacaine at the end of the surgery in the epidural analgesia group, using high performance liquid chromatography-mass spectrometry (LC-MS/MS), as previously described (Nakamura et al., 2008; Ikeda et al., 2010). Briefly, blood was collected from patients at the end of surgery using blood sampling devices with dried heparin (Line Draw Plus; Smith Medical ASD, Inc., Keene, NH), and the plasma was collected by centrifugation and subsequently stored in a 1.5-ml Eppendorf tube at −20°C. All samples were injected into a high-performance liquid chromatograph (CCP&8020 series; Tosoh Corporation, Tokyo, Japan) fitted with a C18 column (ODS-100Z, 20 mm × 50 mm, particle size 5 m; Tosoh Corporation). Levobupivacaine in plasma was extracted with methanol, and ropivacaine was used as an internal standard. Analysis was performed using 4000 Qtrap tandem mass spectrometer (Applied Biosystems, Foster City, CA), and the peak area ratios of levobupivacaine to ropivacaine were used to calculate the plasma concentrations based on least-squares regression of calibrators (10–1,000 ng/ml) included in each run.

### A prospective cohort analysis with 5 ml/h epidural levobupivacaine

Another 30 patients were enrolled to investigate the dose-dependent effect of epidural levobupivacaine with a continuous dose of 5 ml/h (12.5 mg/h) during surgery. This was a prospective, single-center cohort study, which was approved by the Osaka City University Graduate School of Medicine Institutional Review Board, and was registered prior to patient enrollment at University hospital Medical Information Network Center (UMIN000041518, Principal investigator: Kotaro Hori, Date of registration: 22 August 2020). All patients provided written informed consent after being provided a complete description of the study protocol.

Perioperative QTc changes were measured using the same method as above, except for the continuous dose of levobupivacaine without a control group (i.e., there was no “No Epidural Analgesia” group). Similar to the analysis of the above randomized study, linear regression models were used to determine the possible influence of perioperative factors that were previously shown to affect the postoperative QTc interval (Nagele et al., 2012). The plasma concentration of levobupivacaine and electrolytes was measured at the end of surgery.

### Data analysis

The sample size was calculated using G*power software (version 3.1, Heinrich Heine University Duesseldorf, Germany) to detect a mean difference of 20 ms of the QTc interval between the two groups and 30 ms of standard deviation with the Mann–Whitney rank-sum test (*α* = 0.05, *β* = 0.20) (Nakao et al., 2010; Nagele et al., 2012). Since the calculation generated an estimate of 28 patients required for each group, we elected to enroll 60 patients in the randomized study. ECG measurements were manually analyzed using the hard copy paper of ECG by two investigators who were blinded to the allocated groups, and Fridericia’s formula [QTc = QT·(RR)^−1/3^, RR: interval between two QRS complexes] was used to calculate the QTc interval (U.S. Food and Drug Administration, 2005; Nagele et al., 2012). The end of the T wave was identified by the tangent methods, and QT interval was measured by averaging 3 beats (Locati et al., 2017). The U wave was excluded if it is separate or smaller than T wave, but if the U wave was merging into the T wave, QTU interval was measured as QT interval. Additional statistical analyses were performed using SigmaPlot (Systat Software Inc., Chicago, IL). Because the statistical distribution of perioperative QTc intervals was previously reported to be skewed (Johnston et al., 2013), postoperative QTc interval and HR between the two groups was compared using the Mann–Whitney rank-sum test. ANOVA on ranks was used in sub-group analysis for the level of thoracic epidural analgesia, followed by Dunn’s test for multiple comparisons. The correlation coefficient between the plasma concentration of levobupivacaine and postoperative QTc was calculated using a linear regression model. Changes from baseline to postoperative QTc and HR in the population of the cohort study were evaluated using signed rank test. Data are presented as median [interquartile range (IQR)]. No imputation was performed for missing values on the individual variables. A *p*-value < 0.05 was considered statistically significant.

## Results

### Patient characteristics

**FIGURE 1:**
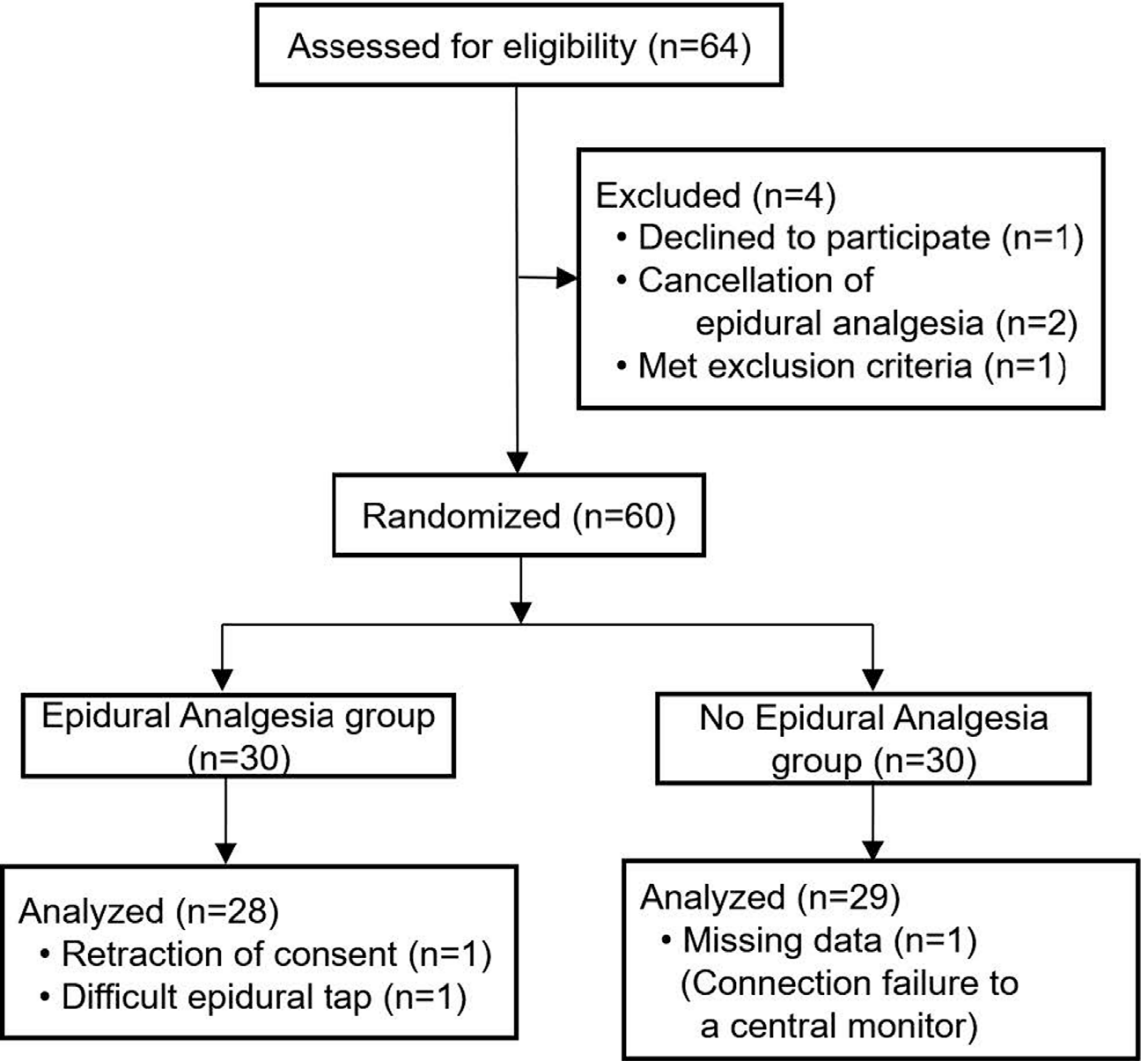
Enrollment, randomization, and analysis for the randomized study with or without epidural analgesia.

Of the 64 patients assessed for eligibility from March 2014 through December 2018, a total of 60 patients undergoing major non-cardiac surgery were enrolled in the randomized study, of whom 57 were assessed for ECG changes during surgery (Figure 1). A total of 28 patients were analyzed as the epidural analgesia group who received epidural levobupivacaine during surgery, and 29 patients were analyzed as the no epidural analgesia group who did not receive any epidural drugs until the end of surgery. Patient characteristics at baseline are shown in Table 1. The median preoperative QTc interval was 402 ms (IQR, 384–424) in the total population, and did not differ between the two groups. Aside from epidural drug administration, patients in the two groups received similar intraoperative care (Table 2).

**TABLE 1:** Preoperative patient baseline.

Characteristic	Total (*n* = 57)	No epidural analgesia (*n* = 29)	Epidural analgesia (*n* = 28)
Age, yr, median (IQR)	71 (63–76)	72 (65–76)	70 (62–76)
Sex, male (%)	32 (56.1)	16 (55.2)	16 (57.1)
Sex, female (%)	25 (43.9)	13 (44.8)	12 (42.9)
Body mass index, kg/m^2^, median (IQR)	23 (21–25)	22 (20–24)	24 (21–25)
ASA class, *N* (%)			
I	4 (7.0)	1 (3.4)	3 (10.7)
II	53 (93.0)	28 (96.6)	25 (89.3)
Laboratory data			
Preoperative QTc, ms, median (IQR)	402 (384–424)	402 (383–424)	402 (388–429)
K^+^, mEq/L, median (IQR)	4.3 (4.1–4.5)	4.3 (4–4.6)	4.4 (4.1–4.5)
Patient medical history^a^, *N* (%)			
Coronary artery disease	4 (7.0)	2 (6.9)	2 (7.1)
Stroke	2 (3.5)	1 (3.4)	1 (3.6)
Diabetes	17 (29.8)	9 (31.0)	8 (28.6)
Preoperative chronic medication, *N* (%)			
Antiarrhythmic	3 (5.3)	1 (3.4)	2 (7.1)
Antipsychotic, Antidepressant	1 (1.8)	1 (3.4)	0 (0)

^a^ Patient medical history was obtained by medical record review at preoperative assessment clinic.

IQR, interquartile range; ASA, American Society of Anesthesiologists; QTc, corrected QT interval.

**TABLE 2:** Intraoperative care measures.

	No epidural analgesia (*n* = 29)	Epidural analgesia (*n* = 28)	Difference^a^ (95% CI)
Surgical time, min, median (IQR)	224 (149–310)	265 (141–334)	−27 (−104 to 49)
Type of surgery, *N* (%)			
Gastroenterological	13 (44.8)	12 (42.9)	2.0 (−23.8–27.7)
Hepato-Biliary-Pancreatic	8 (27.6)	10 (35.7)	−8.1 (−16.0–32.3)
Respiratory	8 (27.6)	5 (17.9)	9.7 (−12.1–31.5)
Urology	0 (0)	1 (3.6)	−3.4 (−10.1 to 3.3)
Medication of interest, median (IQR)			
Sevoflurane, ml in total	73 (49–113)	77 (50–115)	−2 (−26 to 23)
Propofol, mg in total	100 (80–100)	100 (80–130)	−7 (−20 to 5)
Fentanyl, μg in total	200 (100–300)	150 (100–200)	42 (−31–115)
Remifentanil, mg in total	3.28 (1.99–4.41)	2.07 (1.23–3.41)	0.61 (−0.73–1.95)
Rocuronium, mg in total	90 (70–112)	90 (60–110)	0.6 (−21–23)
Ephedrine, mg in total	15 (10–25)	16 (10–25)	0 (−6 to 5)
Variables at extubation, median (IQR)			
Body temperature, °C^b^	36.3 (36–36.9)	36.4 (36.0–37.0)	−0.1 (−0.4–0.5)
K^+^, mEq/L^c^	3.8 (3.5–4.0)	3.7 (3.5–4.0)	0.1 (−0.1–0.3)
Ca^2+^, mEq/L^c^	1.16 (1.12–1.19)	1.13 (1.11–1.17)	0.02 (−0.01–0.06)

^a^ The difference for continuous variables is shown in mean, and that for categorical variables is shown in the absolute difference of percentage points.

^b^ Data regarding body temperature was missing for 1 patient (3.6%) in “No Epidural Analgesia” group.

^c^ Data regarding K^+^ and Ca^2+^ were missing for 3 patients (10.3%) in “No Epidural Analgesia” and 2 patients (7.1%) in “Epidural Analgesia” group.

IQR, interquartile range; QTc, corrected QT interval.

### Postoperative QTc interval: primary outcome

**FIGURE 2:**
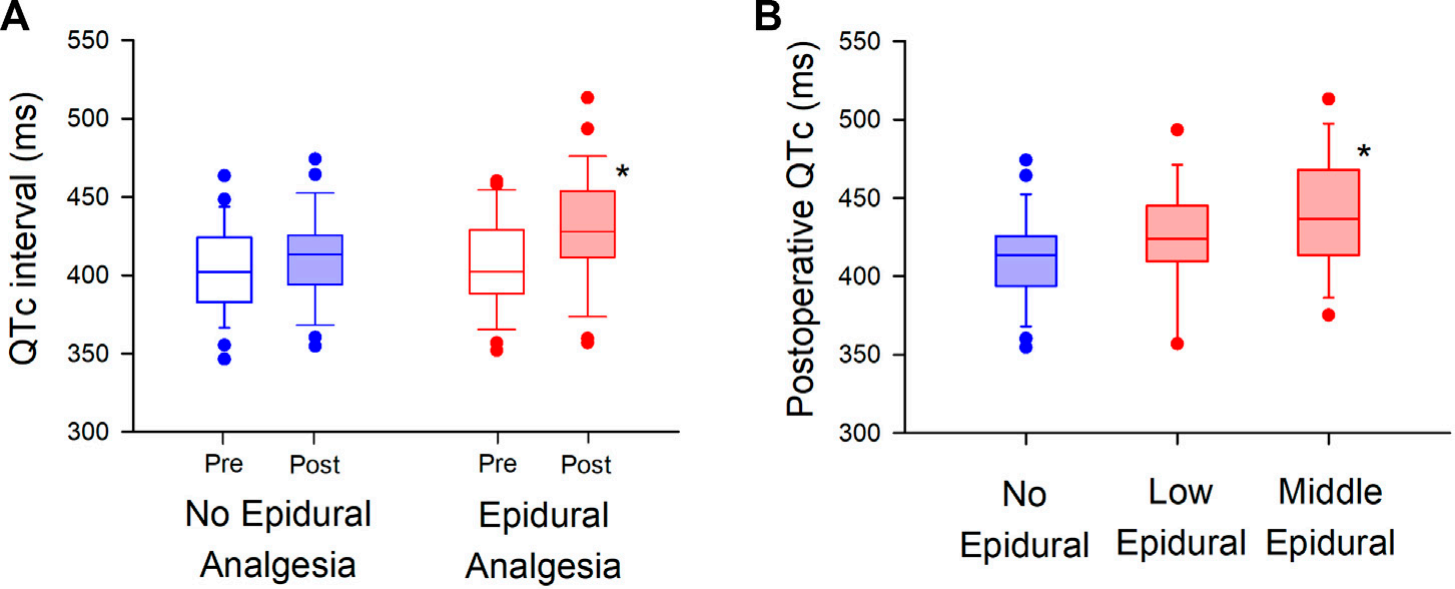
Randomized comparison of QTc interval with or without epidural analgesia in major non-cardiac surgery **(A)**. Postoperative QTc interval is compared between the “No Epidural Analgesia” (left panel, blue-filled box) and “Epidural Analgesia” (right panel, red-filled box) groups, in which epidural 0.25% levobupivacaine at 3 ml/h was administered only in the “Epidural Analgesia” group. The box-and-whisker plots show the medians (middle horizontal lines) and interquartile ranges (boundaries of the box) and ranges. Whisker caps are set at 10% and 90% of the data, and outliers shown are the values lower or higher than the caps. **(B)**. Sub-group analysis is performed in the level of thoracic vertebrae of epidural analgesia (“Low Epidural”; Th 9–12, “Middle Epidural”; Th 5–8). Postoperative QTc intervals are compared in the “No Epidural Analgesia” (left, blue), “Low Epidural” (middle, red), and “Middle Epidural” (right, red) groups. *p < 0.05.

The median postoperative QTc interval in the epidural analgesia group was significantly prolonged compared to that in the no epidural analgesia group [413 ms (IQR, 394–426)] vs. 428 ms (IQR, 411–454), *p* = 0.02, Figure 2A). The median postoperative heart rate (HR) between the groups was not significantly different [83 bpm: beats per minute (IQR, 77–96) vs. 88 bpm (IQR, 75–102), *p* = 0.6]. The interobserver relative error of QTc measurements was 1.5% (IQR, 1.0–2.6). After adjustment for prespecified covariates with the use of multivariable generalized estimating equations, the mean difference in the postoperative QTc interval with or without epidural analgesia was 23 ms [95% confidence interval (CI), 6–40] (*p* = 0.01, Table 3).

**TABLE 3:** Multivariable regression model for postoperative QTc interval.

	Mean difference (ms)	95% confidence interval	*p*-value
Age, yr	0.7	−0.2 to 1.6	0.14
Female sex	0.1	−18.1 to 18.3	0.99
Epidural analgesia	23.2	5.6 to 39.8	0.01
Sevoflurane, ml in total	−0.1	−0.3 to 0.1	0.36
Ephedrine, mg in total	−0.3	−1.2 to 0.5	0.44

QTc, corrected QT interval.

### Exploratory and *post-hoc* outcomes

In exploring the effect of epidural analgesia on the level of thoracic vertebrae of the inserted catheters (Figure 2B), we found that the median postoperative QTc interval with the middle level of thoracic epidural analgesia [437 ms (IQR, 414–468) in “Middle Epidural”; Th 5–8] was significantly prolonged compared to that in the no epidural analgesia group, without epidural analgesia (*p* = 0.03, *n* = 13 in “Middle Epidural” and *n* = 29 in “No Epidural Analgesia”). Whereas, the median postoperative QTc interval with low level of epidural analgesia [424 m (IQR, 409–445) in “Low Epidural”; Th 9–12] was not significantly different from that without epidural analgesia (*p* = 0.3, *n* = 15 in “Low Epidural”). There were no significant differences in the median postoperative HR among the groups [86 bpm (IQR, 76–99) in “Middle Epidural” and 83 bpm (IQR, 77–94) in “Low Epidural”, *p* = 0.7].

**FIGURE 3:**
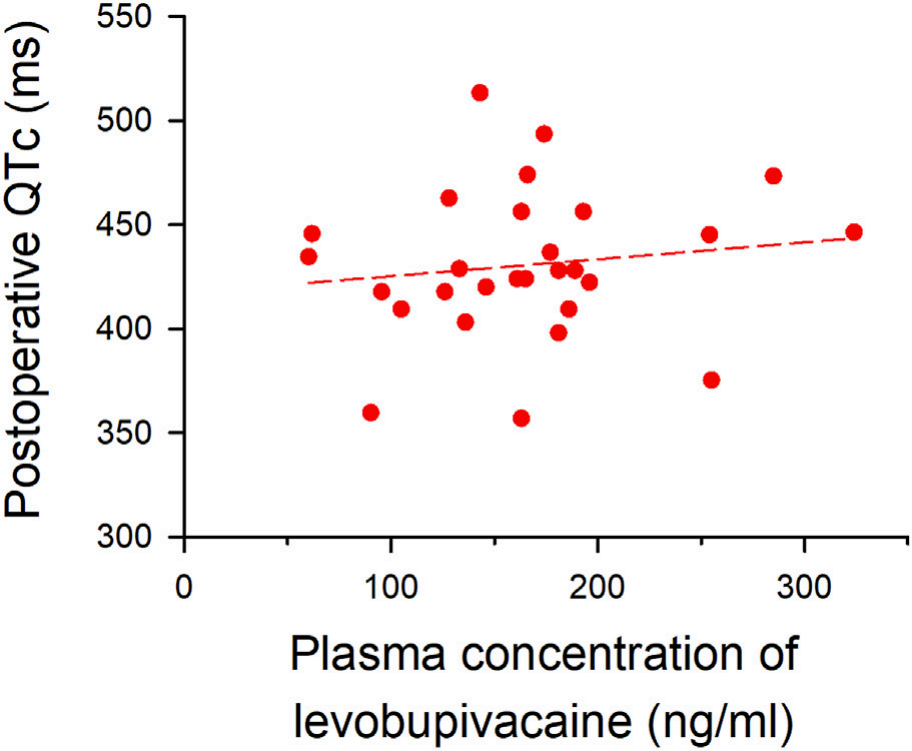
Correlation between plasma concentration of levobupivacaine and postoperative QTc interval. Plasma concentration of levobupivacaine after surgery (*x*-axis) is plotted against postoperative QTc interval (*y*-axis). The formula of the regression line is Y = 417.23928 + (0.081346 × X) (short dash line), and the correlation coefficient (R) was 0.14 (*p* = 0.5).

The median plasma concentration of levobupivacaine at the end of surgery, measured by the LC-MS/MS system, was 164 ng/ml (IQR, 131–188) in the epidural analgesia group. Linear regression analysis was used to evaluate the correlation between plasma levobupivacaine concentration and postoperative QTc interval, in which the correlation coefficient (*R*) was 0.14, showing a very weak and not significant correlation (*p* = 0.5; Figure 3). No critical arrhythmias were detected during thoracic epidural analgesia in either group.

### A prospective cohort analysis with 5 ml/h epidural levobupivacaine

**FIGURE 4:**
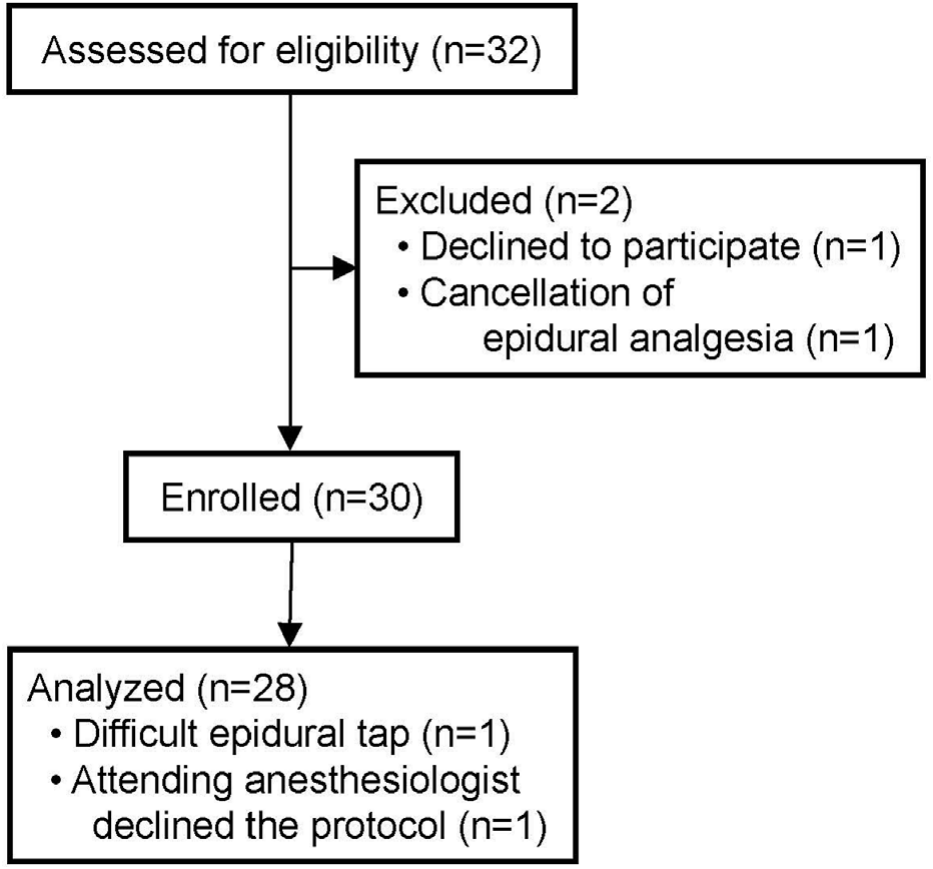
Enrollment and analysis for the prospective cohort study with 5 ml/h epidural levobupivacaine.

**FIGURE 5:**
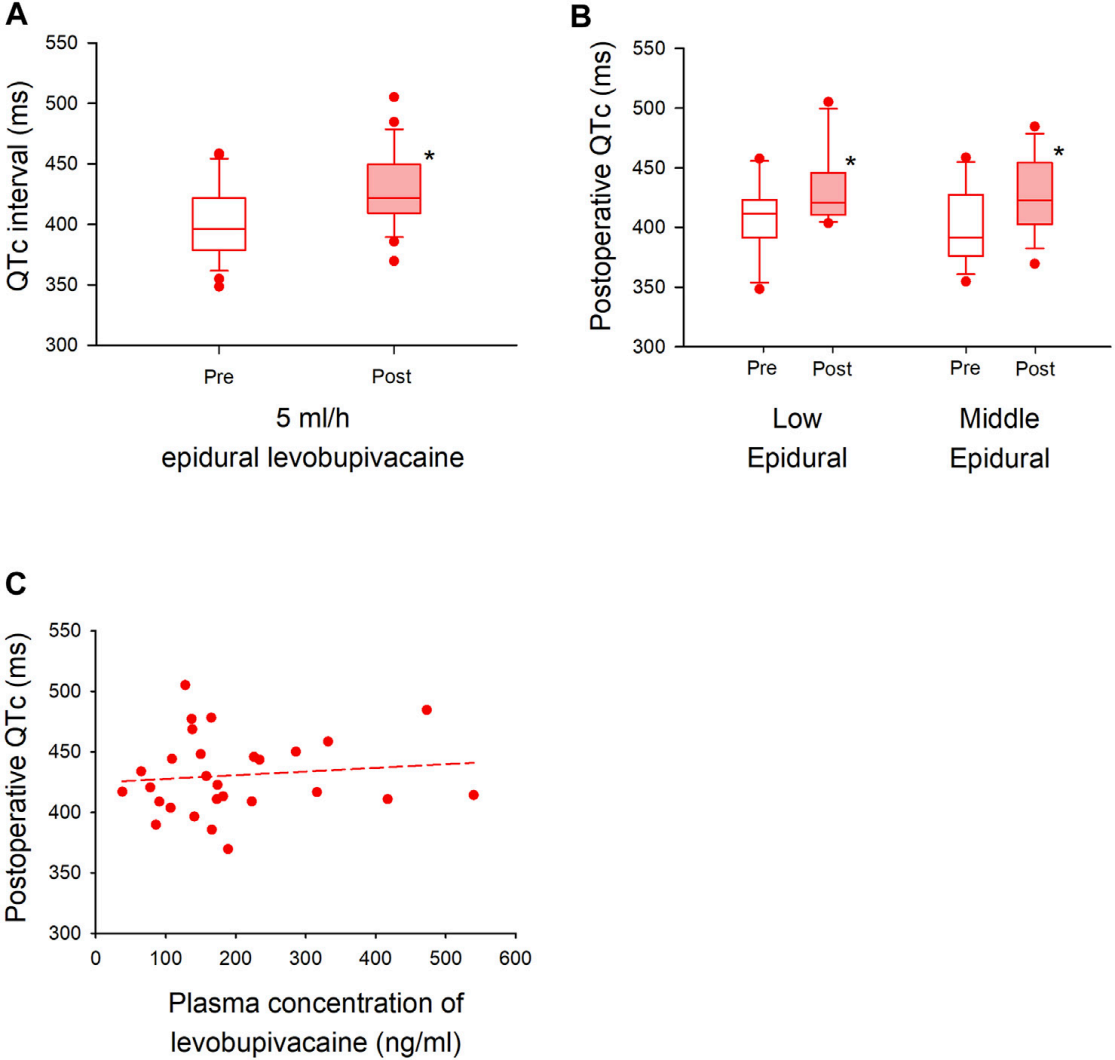
Cohort analysis of QTc interval with higher dose of epidural levobupivacaine in major non-cardiac surgery **(A)**. A QTc interval with 5 ml/h epidural levobupivacaine is shown before (left) and after (right) general anesthesia. The box-and-whisker plots show the medians (middle horizontal lines) and interquartile ranges (boundaries of the box) and ranges. Whisker caps are set at 10% and 90% of the data, and outliers shown are the values lower or higher than the caps. **(B)**. Sub-group analysis is performed in the level of thoracic vertebrae (“Low Epidural”; Th 9–12, “Middle Epidural”; Th 5–8). The QTc interval is compared before and after in each group. **(C)**. Plasma concentration of levobupivacaine with 5 ml/h epidural levobupivacaine (*x*-axis) is plotted against postoperative QTc interval (*y*-axis). The formula of the regression line is Y = 424.52668 + (0.03046 × X) (short dash line), and the correlation coefficient (*R*) was 0.11 (*p* = 0.5). *p < 0.05.

Another 30 patients were enrolled with a continuous dose of 5 ml/h levobupivacaine from August 2020 through January 2022, and 28 of them were assessed for ECG changes during surgery (Figure 4). Patient baseline and perioperative characteristics in the group are shown in Table 4. The median postoperative QTc interval with 5 ml/h epidural levobupivacaine was significantly prolonged compared to baseline [from 396 ms (IQR, 379–422) to 422 ms (IQR, 409–449)], *n* = 28, *p* < 0.00001; Figure 5A). The median postoperative HR was significantly increased compared to baseline [from 71 bpm (IQR, 64–79) to 79 bpm (IQR, 69–90), *p* = 0.04]. Using the prespecified linear regression model, ephedrine dosage was detected as a significant independent variable, and the coefficient was 1.7 ms (95% CI, 0.4–2.9) (*p* = 0.02; Table 5). The median postoperative QTc interval with 5 ml/h epidural levobupivacaine was similar to that with 3 ml/h levobupivacaine in the randomized study (*p* = 0.7, *n* = 28 each; Figures 2A, 5A).

**TABLE 4:** Perioperative data of the cohort analysis with 5 ml/h epidural levobupivacaine.

Preoperative patient baseline	5 ml/h (*n* = 28)
Age, yr, median (IQR)	72 (52–75)
Sex, male/female (%/%)	17/11 (60.7/39.3)
Body mass index, kg/m^2^, median (IQR)	23 (20–25)
ASA class, *N* (%)	
I	2 (7.1)
II	26 (92.9)
Laboratory data	
Preoperative QTc interval, ms, median (IQR)	396 (379–422)
K^+^, mEq/L, median (IQR)	4.2 (3.9–4.5)
Patient medical history, *N* (%)	
Coronary artery disease	1 (3.6)
Stroke	0 (0)
Diabetes	6 (21.4)
Preoperative chronic medication, *N* (%)	
Antiarrhythmic	1 (3.6)
Antipsychotic, Antidepressant	0 (0)
Intraoperative care measures	
Surgical time, min, median (IQR)	210 (159–336)
Type of surgery, N (%)	
Gastroenterological	7 (25.0)
Hepato-Biliary-Pancreatic	6 (21.4)
Respiratory	15 (53.6)
Medication of interest, median (IQR)	
Sevoflurane, ml in total	71 (50–90)
Propofol, mg in total	100 (80–120)
Fentanyl, μg in total	100 (50–200)
Remifentanil, μg in total	2.33 (1.40–3.00)
Rocuronium, mg in total	98 (80–110)
Ephedrine, mg in total	9 (1–19)
Variables at extubation, median (IQR)	
Body temperature, °C	36.8 (36.1–37.4)
K^+^, mEq/L	3.8 (3.4–4.0)
Ca^2+^, mEq/L	1.16 (1.14–1.20)

^a^ Patient medical history was obtained by medical record review at preoperative assessment clinic.

^b^ Data regarding body temperature was missing for 1 patient (3.6%).

^c^ Data regarding K^+^ and Ca^2+^ were missing for 2 patients (7.1%).

IQR, interquartile range; ASA, American Society of Anesthesiologists; QTc, corrected QT interval.

**TABLE 5:** Multivariable regression model for postoperative QTc interval of the cohort study.

	Mean difference (ms)	95% confidence interval	*p*-value
Age, yr	−0.04	−0.86 to 0.77	0.92
Female sex	13.25	−10.13 to 36.62	0.29
Sevoflurane, ml in total	0.21	−0.12 to 0.54	0.22
Ephedrine, mg in total	1.67	0.41 to 2.92	0.02

QTc, corrected QT interval.

At the level of the inserted epidural catheters with 5 ml/h levobupivacaine, the median postoperative QTc interval both in the middle level (Th 5–8) and low level (Th 9–12) of epidural analgesia was significantly prolonged compared to baseline [low level; from 411 ms (IQR, 392–423) to 420 ms (IQR, 411–446), *n* = 11, *p* = 0.005, middle level; from 392 ms (IQR, 376–428) to 423 ms (IQR, 403–454), *n* = 17, *p* = 0.007; Figure 5B]. There were no significant changes in the median HR in both groups [low level; from 71 bpm (IQR, 58–75) to 83 bpm (IQR, 69–90), *p* = 0.07, middle level; from 75 bpm (IQR, 68–83) to 75 bpm (IQR, 66–93), *p* = 0.3]. The median plasma concentration of levobupivacaine with 5 ml/h epidural analgesia was 166 ng/ml (IQR, 114–232), and the correlation coefficient between plasma levobupivacaine concentration and postoperative QTc interval was 0.11, showing a very weak and not significant correlation (*p* = 0.5) (Figure 5C). No critical arrhythmias were detected during epidural analgesia also in this cohort study.

## Discussion

Our randomized study showed that thoracic epidural analgesia with 3 ml/h levobupivacaine prolonged QTc interval after general anesthesia in major non-cardiac surgery. Using multiple regression analysis for perioperative factors known to affect QTc interval, epidural analgesia was found to be an independent variable for the postoperative QTc prolongation, and the mean difference of the QTc interval with or without epidural analgesia was 23 ms after the adjustment. Our prospective cohort study also showed that 5 ml/h epidural levobupivacaine prolonged postoperative QTc interval than preoperative baseline. Plasma concentrations of levobupivacaine after surgery were very low (ng/ml level) both with 3 and 5 ml/h epidural administration of levobupivacaine during surgery. Epidural analgesia is a common modality for the management of surgical pain; therefore, it is important to know that epidural analgesia could prolong QTc interval after major non-cardiac surgery.

There are several factors in perioperative period that are known to affect cardiac repolarization; thus, the postoperative QTc interval would be a result of the cumulative effects of such factors. Nagele et al. (2012) previously showed that QTc prolongation after general anesthesia was frequent and the perioperative factors that had a large effect on QTc prolongation were isoflurane, methadone, ketorolac, cefoxitin, zosyn, unasyn, epinephrine, ephedrine, and calcium. Similar to their results, in our cohort population with epidural analgesia of 5 ml/h levobupivacaine, ephedrine was shown to be an independent factor for the postoperative QTc prolongation (Table 5). In contrast, in our randomized study to compare the postoperative QTc interval with or without 3 ml/h epidural levobupivacaine, we found epidural analgesia to be an only independent variable for the postoperative QTc prolongation, which had a much larger effect than ephedrine (Table 3).

Interestingly, our data showed that epidural analgesia could independently prolong QTc interval after surgery, and the mechanism of this prolongation is also of great interest. One possible mechanism is reflective stimulation of cardiac sympathetic nerves by blocking the neighboring or part of the cardiac nerves with epidural analgesia. A previous animal study showed that epidural block to the median level of Th 8 increased activity of cardiac sympathetic nerves derived from Th 1–4, and the activation of the nerves was abolished with a higher epidural block to the median level of cervical vertebrae 8 or the surgical denervation of the carotid sinus and vago-aortic nerves (Taniguchi et al., 1997). In our sub-group analysis of the randomized study, a middle level of thoracic epidural analgesia (Th 5–8) with 3 ml/h levobupivacaine significantly prolonged postoperative QTc interval as compared to that in the no epidural analgesia group, whereas a low level of thoracic epidural (Th 9–12) did not (Figure 2B). Using a higher dose of levobupivacaine (5 ml/h), our cohort analysis showed that both low and middle levels of thoracic epidural significantly prolonged QTc intervals compared to baseline (Figure 5B). Considering our results and the above-mentioned studies (Taniguchi et al., 1997; Owczuk et al., 2005; Owczuk et al., 2009; Bourke et al., 2010; Dogan et al., 2014), the postoperative QTc interval with epidural analgesia seems to depend on the level of the blockade in the thoracic vertebrae, suggesting a possible effects of cardiac sympathetic nerves on the mechanism of QTc prolongation.

Another possible mechanism for postoperative QTc prolongation is the systemic effect of epidurally administered levobupivacaine, which is absorbed into the small blood vessels abundant around the epidural space. The molecular mechanism of QTc prolongation have not been fully elucidated, but since QT interval is the time from the ventricular depolarization to the end of repolarization, it is theoretically related to the effects of sodium, potassium, and calcium channels. Especially, HERG potassium channels are thought to play an important role in QTc prolongation (Locati et al., 2017). As levobupivacaine in the blood blocks cardiac sodium, calcium as well as HERG potassium channels, it can directly prolong the QTc interval (Graf et al., 1997; Gonzalez et al., 2002). Bardsley et al. (1998) studied the cardiovascular effects of systemic levobupivacaine with intravenous infusion in volunteers to the dose at which the volunteers showed central nervous system symptoms, or the upper limit of 150 mg. In their study, QTc intervals did not significantly increase with the drug dosage, and the mean maximum plasma concentration of levobupivacaine was 2.62 μg/ml. A previous report measuring plasma levobupivacaine concentration with epidural analgesia showed that 5–10 ml/h continuous administration of epidural 0.2% levobupivacaine could lead to a higher plasma concentration than 2.5 μg/ml (Lauprecht et al., 2011). However, in our study, 3–5 ml/h of 0.25% levobupivacaine was used, and our median concentrations of levobupivacaine at the end of surgery were much lower: 164 ng/ml (IQR, 131–188) with 3 ml/h and 166 ng/ml (IQR, 114–232) with 5 ml/h (Figures 3, 5C). Considering our measured plasma concentration of levobupivacaine and correlation coefficient to the postoperative QTc interval, systemic effects of epidurally administered levobupivacaine were unlikely to affect the QTc interval in our study settings.

This study has several potential limitations. First, it is interesting to know that epidural analgesia could postoperatively increase the mean QTc interval by 23 ms after adjustment of perioperative factors, but the relationship between the QTc prolongation and lethal arrhythmia is still unclear because of rare incidence of perioperative TdP (Nagele et al., 2012; Johnston et al., 2013). To our knowledge, there is a human case report of perioperative TdP with epidural analgesia, in which the only potential cause of the TdP was epidural administration of bupivacaine, a racemic mixture of levobupivacaine (Broka et al., 1999). In the studies of newly developed drugs to evaluate drug-induced QTc prolongation for approval process, QTc prolongation is generally defined as more than 10 ms increase (Roden, 2016), which is considered to have some arrhythmogenic risks with the drugs. Actually, several drugs have been withdrawn from the market because they cause TdP with QTc prolongation for around 10 ms (Pratt et al., 1996; Khongphatthanayothin et al., 1998). Therefore, we believe that our data of QTc prolongation with epidural analgesia is the important finding for better safety of perioperative analgesia. Second, the patients in our study had a three-lead ECG to assess cardiac repolarization with ⅠⅠ-lead, not 12-lead ECG. Twelve-lead ECG would have allowed for a more detailed analysis. The most appropriate lead of ECG to evaluate cardiac repolarization has not been established, but ⅠⅠ-lead is a commonly used lead to measure QTc interval (U.S. Food and Drug Administration, 2005), and is also a standard monitoring lead during anesthesia. Thus, we think that our data are clinically very useful because the postoperative QTc prolongation was detectable in ⅠⅠ-lead, which means it is possible to evaluate the risk of delayed cardiac repolarization with the usual ECG monitoring lead during anesthesia. Third, we measured QTc interval at baseline and postoperative period by averaging 3 beats, which would have allowed us to minimize the short-term beat-to-beat variability of QTc intervals, but not the longer minute-to-minute variability. Future studies by analyzing a large digital ECG database may reveal the influence of the long-term variability on perioperative QTc intervals.

In conclusion, our randomized and prospective cohort studies showed that thoracic epidural analgesia could enhance postoperative QTc prolongation after general anesthesia in patients undergoing major non-cardiac surgery. The mechanism of QTc prolongation is possibly caused by blocking the neighboring or part of the cardiac sympathetic nerves, rather than by systemic effects of epidurally administered levobupivacaine. Although perioperative TdP is a very rare event, epidural analgesia is a commonly used modality to relieve surgical pain, and therefore, postoperative QTc prolongation should be carefully monitored to avoid fatal arrhythmias in perioperative management.

## Data Availability

The original contributions presented in the study are included in the article/supplementary material, further inquiries can be directed to the corresponding author.
